# Automated thermal imaging for the detection of fatty liver disease

**DOI:** 10.1038/s41598-020-72433-5

**Published:** 2020-09-23

**Authors:** Rafael Y. Brzezinski, Lapaz Levin-Kotler, Neta Rabin, Zehava Ovadia-Blechman, Yair Zimmer, Adi Sternfeld, Joanna Molad Finchelman, Razan Unis, Nir Lewis, Olga Tepper-Shaihov, Nili Naftali-Shani, Nora Balint-Lahat, Michal Safran, Ziv Ben-Ari, Ehud Grossman, Jonathan Leor, Oshrit Hoffer

**Affiliations:** 1 Neufeld Cardiac Research Institute, Sackler Faculty of Medicine, Tel Aviv University, 6997801 Tel Aviv, Israel; 2 Tamman Cardiovascular Research Institute, Leviev Heart Center, Sheba Medical Center, 52621 Tel Hashomer, Israel; 3 Department of Industrial Engineering, Tel-Aviv University, 6997801 Tel Aviv, Israel; 4 School of Medical Engineering, Afeka Tel Aviv Academic College of Engineering, 6910717 Tel Aviv, Israel; 5 School of Electrical Engineering, Afeka Tel Aviv Academic College of Engineering, 6910717 Tel Aviv, Israel; 6 Pathology Institute, Sheba Medical Center, 52621 Tel Hashomer, Israel; 7 Liver Disease Center, Sheba Medical Center, 52621 Tel Hashomer, Israel; 8 Internal Medicine Wing and Hypertension Unit, Sheba Medical Center, 52621 Tel Hashomer, Israel; 9 Sackler Faculty of Medicine, Tel Aviv University, 6997801 Tel Aviv, Israel

**Keywords:** Hepatology, Liver diseases, Medical imaging

## Abstract

Non-alcoholic fatty liver disease (NAFLD) comprises a spectrum of progressive liver pathologies, ranging from simple steatosis to non-alcoholic steatohepatitis (NASH), fibrosis and cirrhosis. A liver biopsy is currently required to stratify high-risk patients, and predicting the degree of liver inflammation and fibrosis using non-invasive tests remains challenging. Here, we sought to develop a novel, cost-effective screening tool for NAFLD based on thermal imaging. We used a commercially available and non-invasive thermal camera and developed a new image processing algorithm to automatically predict disease status in a small animal model of fatty liver disease. To induce liver steatosis and inflammation, we fed C57/black female mice (8 weeks old) a methionine-choline deficient diet (MCD diet) for 6 weeks. We evaluated structural and functional liver changes by serial ultrasound studies, histopathological analysis, blood tests for liver enzymes and lipids, and measured liver inflammatory cell infiltration by flow cytometry. We developed an image processing algorithm that measures relative spatial thermal variation across the skin covering the liver. Thermal parameters including temperature variance, homogeneity levels and other textural features were fed as input to a t-SNE dimensionality reduction algorithm followed by k-means clustering. During weeks 3,4, and 5 of the experiment, our algorithm demonstrated a 100% detection rate and classified all mice correctly according to their disease status. Direct thermal imaging of the liver confirmed the presence of changes in surface thermography in diseased livers. We conclude that non-invasive thermal imaging combined with advanced image processing and machine learning-based analysis successfully correlates surface thermography with liver steatosis and inflammation in mice. Future development of this screening tool may improve our ability to study, diagnose and treat liver disease.

## Introduction

Non-alcoholic fatty liver disease (NAFLD) represents the hepatic manifestation of metabolic syndrome and is considered the leading cause of chronic liver disease^1^. NAFLD encapsulates a spectrum of progressive liver pathologies, ranging from simple steatosis to non-alcoholic steatohepatitis (NASH), fibrosis, and cirrhosis^2^. Approximately one quarter of the world's population is diagnosed with simple liver steatosis and this number is projected to increase dramatically with the upward trend of obesity worldwide^1^.

Currently, a liver biopsy is required to confirm the presence of NASH. Moreover, accurately assessing liver inflammation and fibrosis using non-invasive tests remains challenging^3^. Cost-effective monitoring strategies of liver pathology are critical for both clinical decision-making and research of new therapeutic targets for NAFLD associated diseases.

Thermal infrared imaging is a non-invasive tool that does not require exposure to ionizing radiation^4^. Recent developments of commercially available thermal imaging products have attracted considerable attention for use of infrared thermography in biomedical imaging^5–7^. We have recently demonstrated that non-invasive thermal imaging combined with advanced image processing algorithms and machine learning-based analysis can correlate surface thermography with structural changes in internal organs of mice such as the heart^8^. The anatomical proximity of the liver to the skin surface and its vast vascular bed might enable the detection of changes in surface thermography through the use of non-invasive thermal imaging^9^.

**Figure 1:**
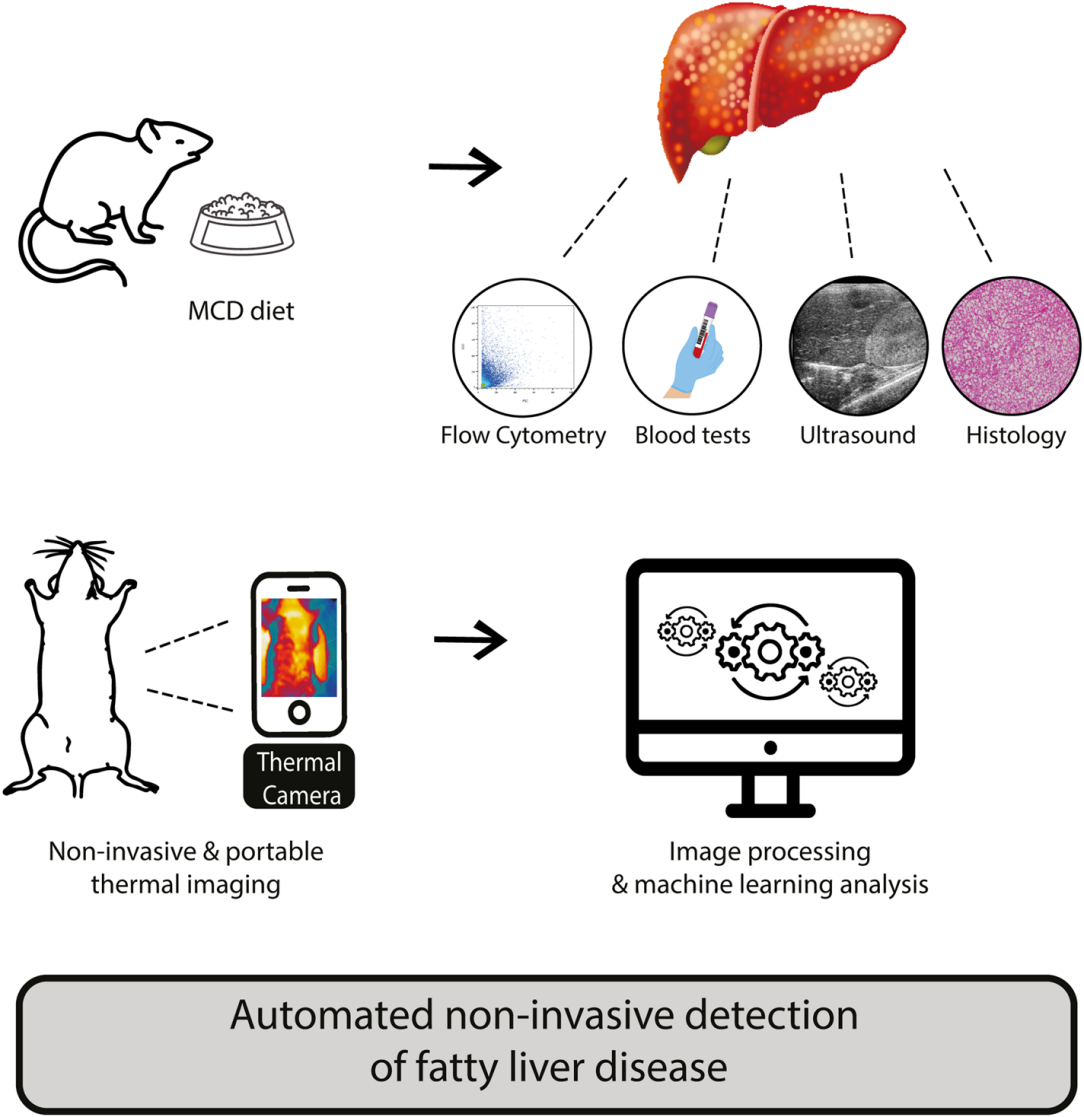
Research design. A graphic scheme describing our research design to develop a new, quick and easy-to-handle tool to image fatty liver disease. (Drawings adapted from BigMouse/Shutterstock.com; Julia Pankin/Shutterstock.com; nexusby/Shutterstock.com; grmarc/Shutterstock.com; bsd/Shutterstock.com; unlimicon/thenounproject.com; TheIcon Z/thenounproject.com).

Here, we sought to use a novel thermal image processing algorithm to automatically diagnose and monitor liver steatosis and inflammation in a small animal model of fatty liver disease in mice fed a methionine-choline deficient (MCD) diet. We aim to develop a new cost-effective screening modality for patients with liver steatosis and other animal models of chronic liver disease (Fig. 1).

## Results

### MCD diet induced liver steatosis and inflammation over time

**Figure 2:**
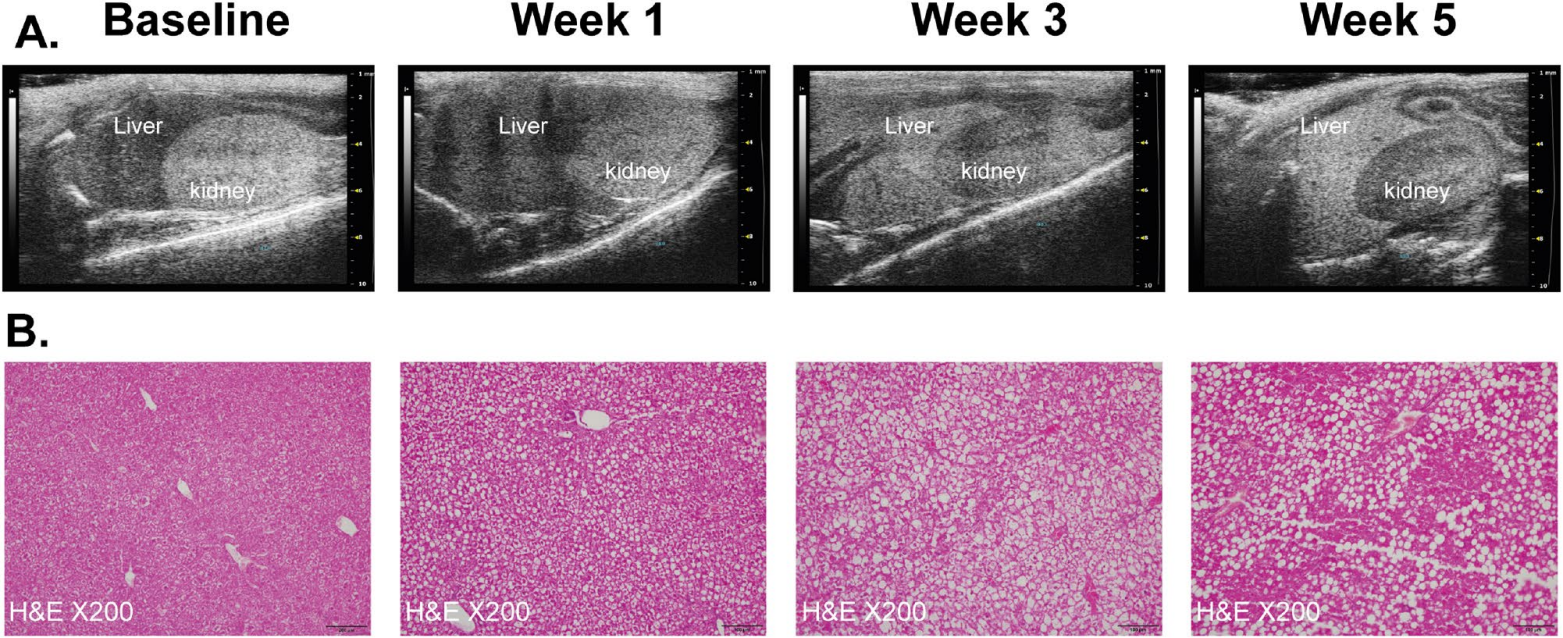
A methionine-choline deficient diet induced liver steatosis and inflammation in mice. **(A)** We performed serial ultrasound studies throughout a 6-week course of MCD diet. Presented are representative pictures of the kidney and liver (labeled). The liver tissue displayed elevated echogenicity over time, a key feature of liver steatosis in ultrasound. Visually, the liver transformed to appear brighter than kidney tissue throughout the course of the diet. **(B)** We dissected livers for histopathological staining at each time point. The H&E staining displayed accumulating fatty infiltrates (white vesicles) over time. *MCD *methionine-choline deficient, *H&E* hematoxylin and eosin.

To assess the ability of our imaging technique to detect liver pathology associated with NAFLD, we first sought to establish a robust model of liver steatosis and inflammation^10,11^. A 6-week course of an MCD diet induced severe liver steatosis, evident in both ultrasound and histopathological evaluation of the liver (Fig. 2A,B). Liver steatosis was present after just one week of MCD-diet, and steatosis increased over time (Fig. 2A,B).

**Figure 3:**
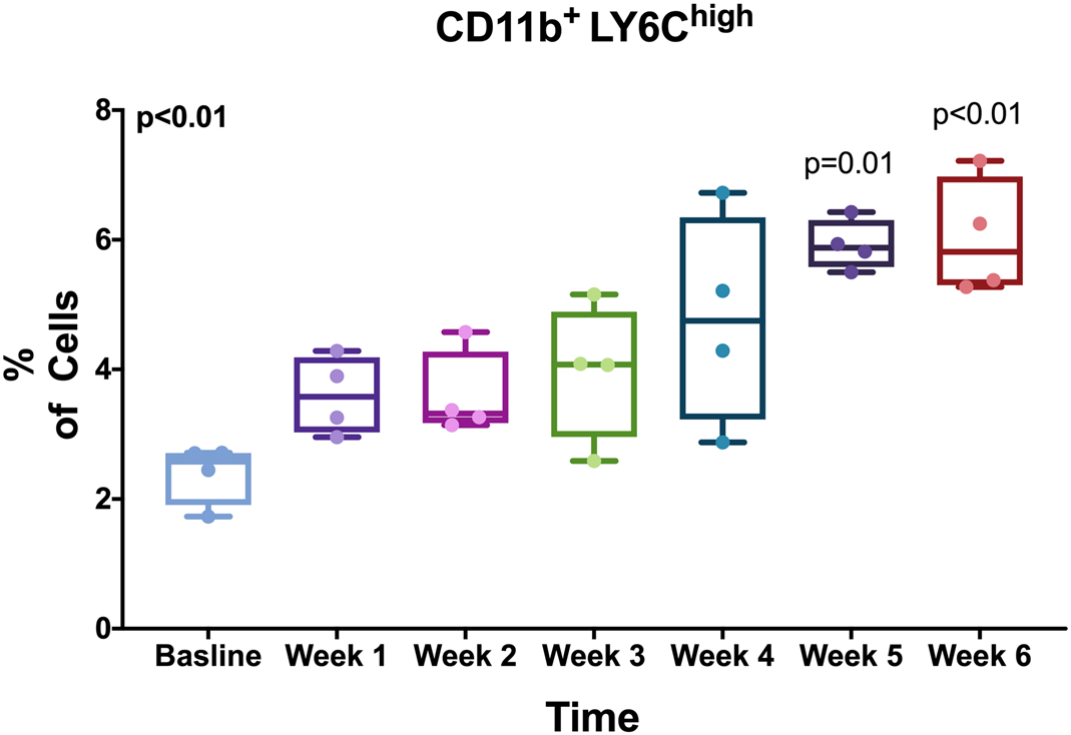
MCD diet increased the levels of pro-inflammatory monocytes in the liver. We dissected livers from mice after each week of MCD diet and measured inflammatory cell surface markers by flow cytometry. We saw an increase in the amount of pro-inflammatory monocytes throughout the progression of the disease (CD11b^+^ LY6C^high^). Displayed is a box-and-whisker plot with individual values. P-values by Kruskal–Wallis and Dunn’s test for multiple comparisons.

Next, we sought to evaluate liver inflammation and characterize the immune cell populations in the liver over time. We measured inflammatory cell surface markers by flow cytometry at baseline and after each week of the MCD diet. MCD diet-fed mice showed a significant increase in monocytes (CD11b^+^ ly6c^+^) in the liver over time. Specifically, we saw a time-dependent increase in the amount of pro-inflammatory monocytes (CD11b^+^ LY6C^high^) in MCD diet-fed mice (Fig. 3).

**Figure 4:**
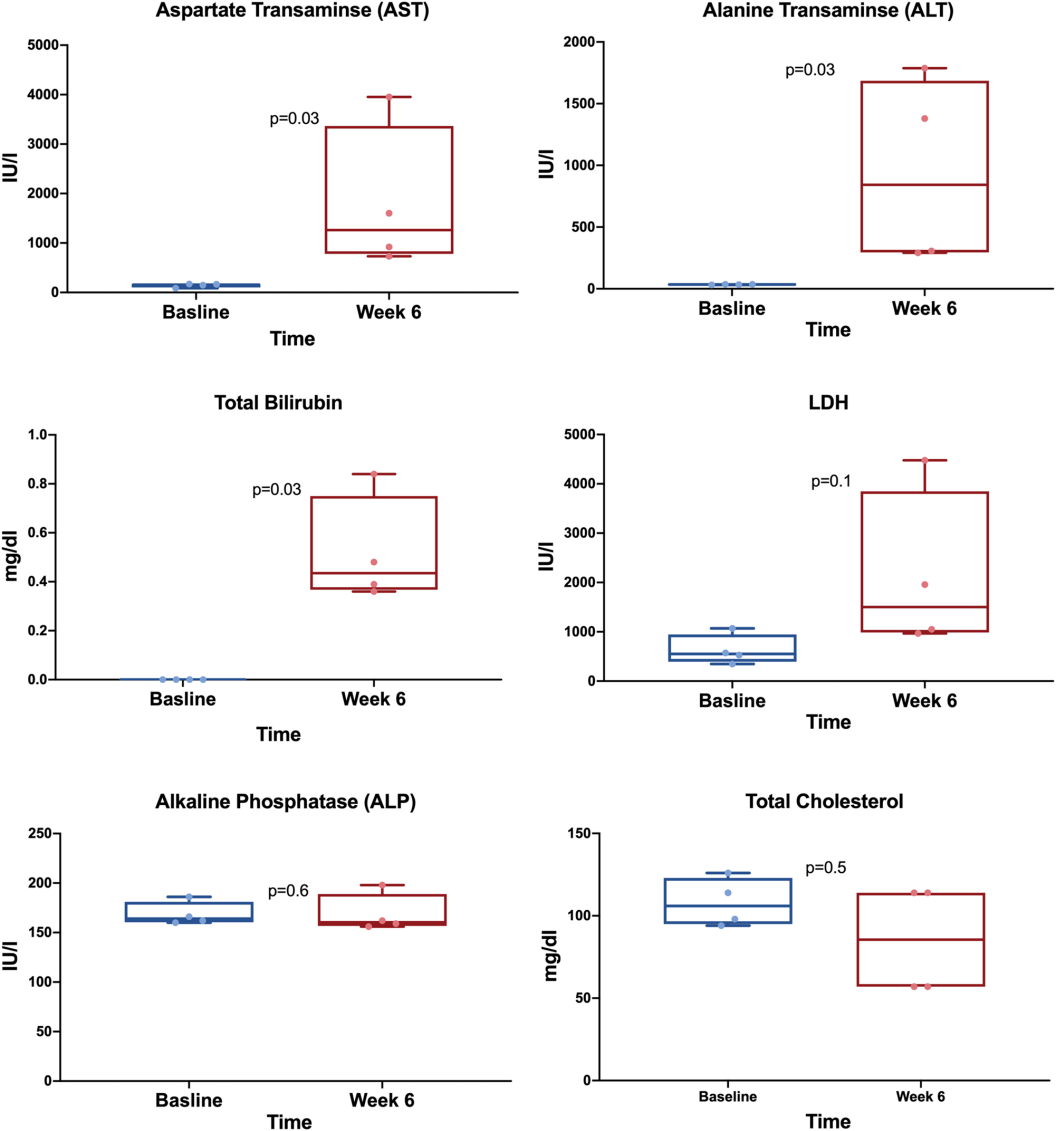
MCD-diet altered blood liver enzymes and lipid profile. We drew blood from the hearts of mice at baseline and after 6 weeks of MCD diet and ran a chemistry panel. The liver enzymes AST and ALT increased from baseline along with total bilirubin and LDH. Total cholesterol levels slightly decreased from baseline concentrations, while ALP concentrations did not change significantly. Displayed are box-and-whisker plots with individual values. P-values by Mann–Whitney test. *MCD *methionine-choline deficient,* ALT *alanine transaminase,* AST *aspartate transaminase*, ALP *alkaline phosphatase, *LDH *lactate dehydrogenase.

Finally, we assessed liver injury and function by measuring serum liver enzymes and lipids. MCD diet-fed mice demonstrated a significant elevation in the liver enzymes aspartate transaminase (AST) and alanine transaminase (ALT) along with increased total bilirubin levels. Lactate dehydrogenase (LDH) levels were also elevated, albeit not statistically significant (Fig. 4). Total cholesterol levels decreased from baseline concentrations, while ALP concentrations did not change significantly.

Taken together, we conclude that an MCD diet for a course of 6 weeks recapitulated key features of fatty liver disease, including significant structural changes, inflammation and impaired liver function.

### Steatosis and inflammation altered the thermal energy emitted by the liver

To determine whether structural changes and inflammation lead to distinct changes in the liver’s surface thermography, we captured intra-abdominal direct thermal images of the liver in situ in live (sedated) mice after 6 weeks of an MCD/regular diet. Thermal images were analyzed by our image processing algorithm and the 9 extracted features were compared between the two groups.

**Figure 5:**
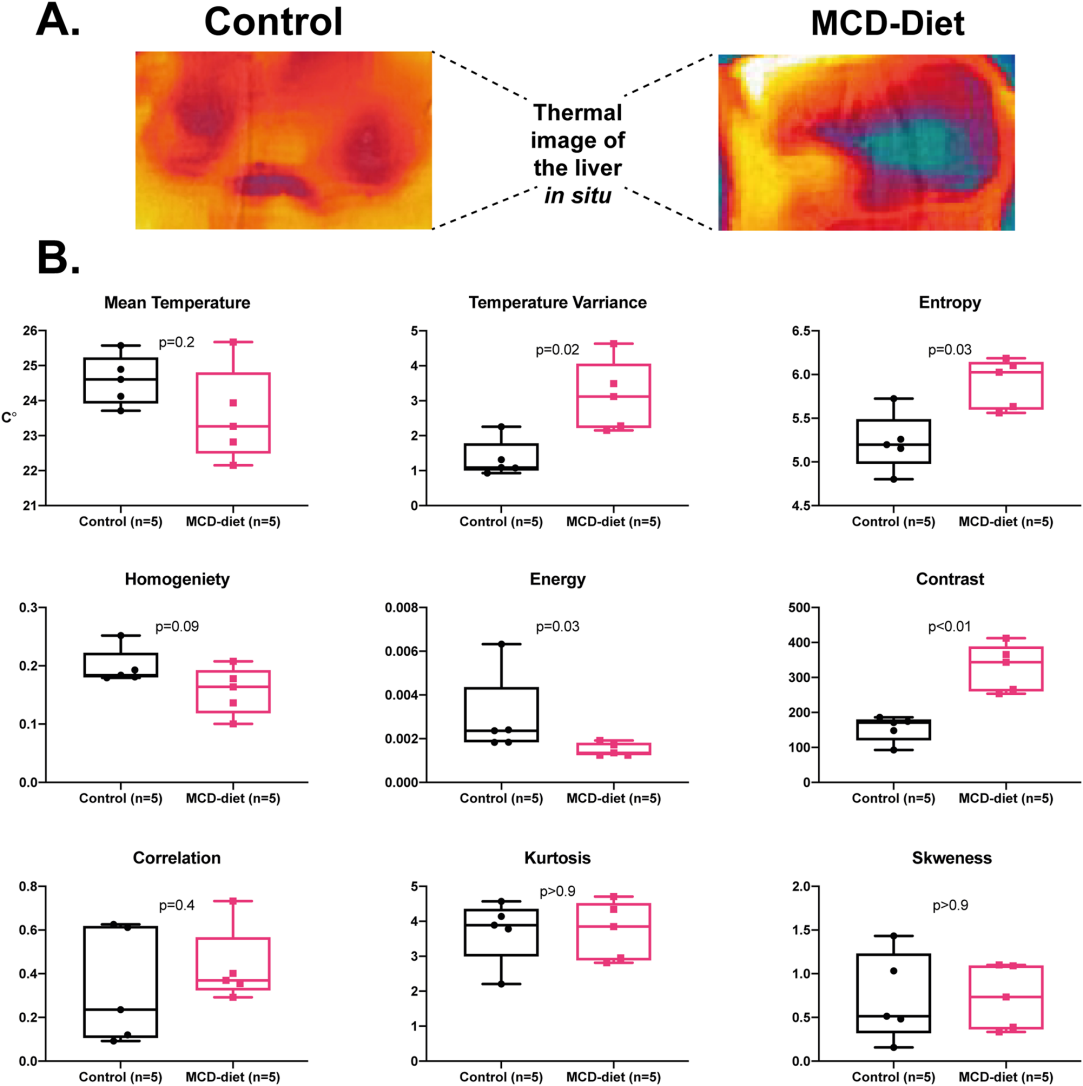
Thermal imaging of livers in situ*.* We captured direct thermal images of the livers in situ in live (sedated) mice fed an MCD vs regular diet for 6 weeks. **(A)** Thermal image of the liver tissue. **(B)** Presented are multiple texture features extracted from the thermal image by our image processing algorithm. Livers of mice fed an MCD diet demonstrated elevated levels of heterogeneity across the thermal image measured by temperature variance, entropy, and contrast, combined with decreased homogeneity and energy values. Displayed are box-and-whisker plots with individual values. P-values by Mann–Whitney test.

Livers of mice fed an MCD diet demonstrated distinct changes in their thermographic profile which indicated elevated levels of heterogeneity across the thermal image. Temperature variance, entropy, and contrast were significantly elevated in MCD diet-fed mice (Fig. 5). Accordingly, both homogeneity and energy values were lower in MCD mice compared with regular diet controls.

Combined with our histopathological and ultrasound findings, these results indicate that the presence of liver steatosis and inflammation is associated with distinct changes in tissue surface thermography, and that these changes can be successfully monitored by thermal infrared imaging.

### Non-invasive thermal image processing detected liver disease status

**Figure 6:**
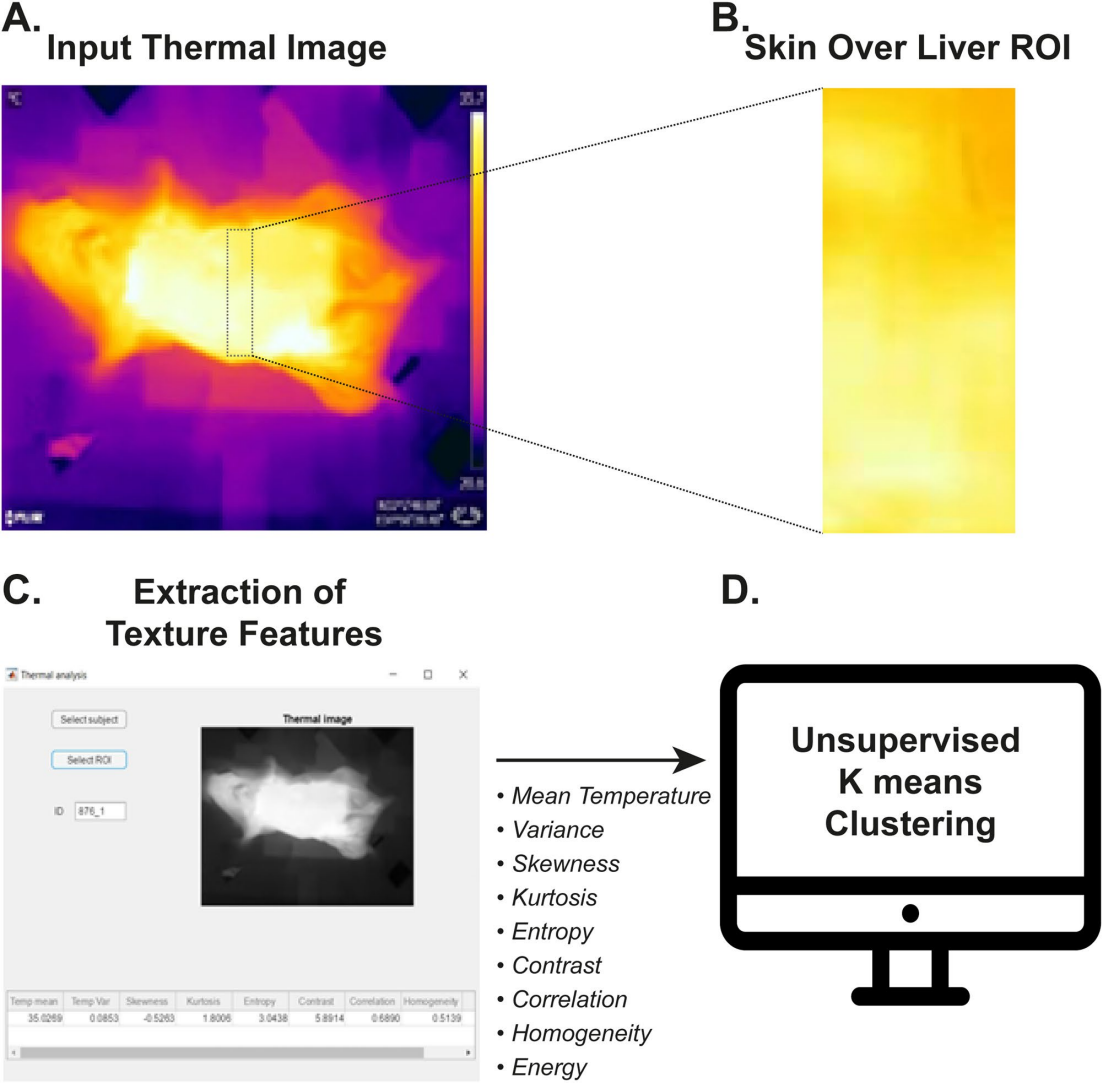
Non-invasive thermal image processing. **(A)** Non-invasive thermal images (IRON scale) of the mice were captured weekly. **(B, C)** Thermal images were processed by our algorithm which extracts multiple features from the selected region of interest (ROI) covering the liver. Displayed are the ROI **(B)** and the Graphical User Interface **(C)** we developed. **(D)** Output parameters were fed as input into a t-SNE dimensionality reduction algorithm, followed by k-means clustering. *t-SNE *t-distributed stochastic neighbor embedding.

Finally, we sought to determine whether the changes in surface thermography of diseased livers could be used to automatically diagnose and monitor liver pathology with non-invasive thermal imaging. Our image processing algorithm measures relative spatial temperature variation across the skin covering the liver. The algorithm extracts 9 features out of each thermal image captured throughout the experiment (Fig. 6).

**Figure 7:**
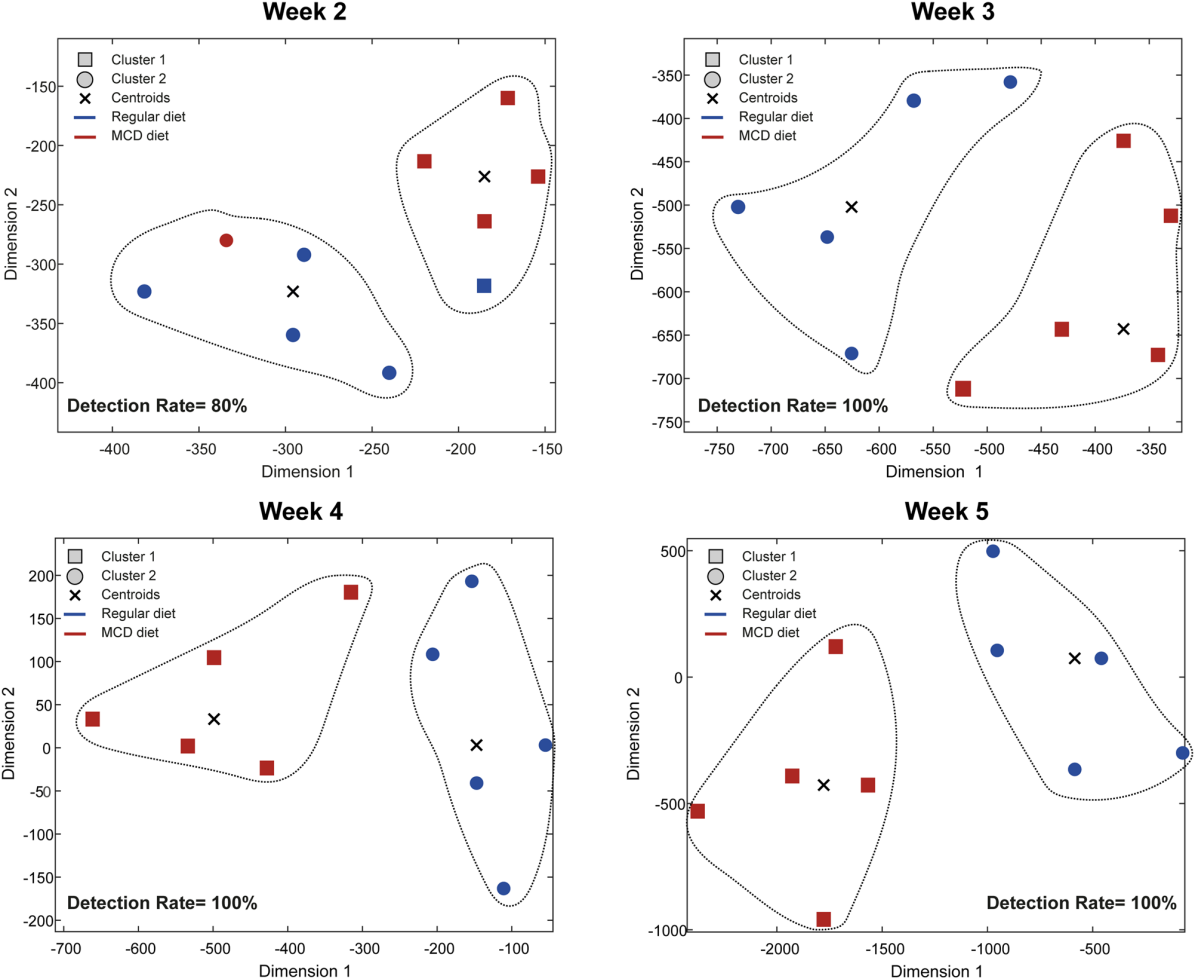
Machine learning-based analysis of non-invasive thermal image processing. t-SNE plots representing the diagnostic yield of our model are presented. During weeks 3, 4, and 5, our algorithm demonstrated a 100% detection rate and clustered 10 of the 10 mice correctly (MCD diet vs regular control diet). *t-SNE *t-distributed stochastic neighbor embedding.

We applied a k-means clustering algorithm to automatically predict the assigned diet (MCD/regular) for each mouse. During week 2 of the experiment, 8 of 10 mice were classified correctly by our model. During weeks 3, 4, and 5, our algorithm demonstrated a 100% detection rate: 10 of the 10 mice were associated to homogeneous clusters of distinct types (MCD/ regular diet). A t-SNE plot representing the diagnostic yield of our model is presented in Fig. 7.

These findings indicate that our image processing tool and machine learning algorithm successfully correlated skin surface thermography with liver pathology.

## Discussion

Our findings suggest, for the first time, that non-invasive thermal imaging could potentially be used to study liver disease in mice. We show that a thermal camera using a novel image processing algorithm and downstream machine learning analysis can automatically distinguish between animals with and without liver pathology. To our knowledge, this is the first report to describe the effect of liver steatosis and inflammation on the liver’s thermal properties in vivo.

Current non-invasive imaging modalities that monitor the progression of steatohepatitis and fibrosis are limited^3^. The potential imaging tool developed here has several critical advantages. First, the capturing of images is simple, quick, and does not require technical training, unlike abdominal ultrasound. Second, our image processing algorithm is compatible with any commercially available thermal camera, making it potentially more affordable and scalable for use in various research settings. Finally, the thermal camera used here, like similar available products, is portable and connects directly to smartphones. Therefore, future development of this model in humans could enable rapid and close monitoring of disease progression and various treatments, with reduced effort and time. This new imaging modality could be especially relevant for out-of-hospital settings.

Attempts to use thermal imaging to monitor liver disease have been previously reported. The majority of these attempts captured thermal images of ex vivo perfused liver samples. Thermal image analysis was able to correlate liver thermography with liver viability in transplanted livers^12^, and identify liver fibrosis and steatosis when combined with chemical imaging techniques^13^ or other advanced spectroscopy methods^14^. Several reports have also described the utility of thermal imaging in monitoring tissue damage during various liver ablation procedures^15–17^.

Our study is the first report that describes the potential use of non-invasive thermal imaging of skin surface thermography to monitor steatohepatitis. Moreover, our use of direct intra-abdominal thermal imaging of the liver in situ in live (sedated) mice, supports our hypothesis that cellular and molecular mechanisms of liver pathology lead to distinct changes in the liver’s emitted thermal energy.

A major strength of our study is the extensive characterization of the mouse model we used to recapitulate key features associated with NAFLD (the MCD diet model), including ultrasound studies, histopathological analysis, flow cytometry, and measurement of serum liver enzymes and lipids.

Non-invasive thermal imaging of the skin surface has already been suggested as an alternative method to assess liver pathology in newborns over 40 years ago^9,18,19^. However, no notable progress towards clinical use has been reported since. Our image processing algorithm and machine learning-based techniques provide an evolved approach to process thermal images, focusing not only on absolute temperature measurement across the tissue, but also on texture parameters of various region of interests. Future clinical studies using our thermal imaging technique are needed in order to identify the most relevant thermal features that correlate with disease progression. Identifying these thermal texture parameters of the skin covering the liver could possibly be used to calculate new risk scores for patients with NAFLD. Implementing this technique could also lead to improvements in thermal-based imaging in other fields of medicine.

Our study is limited by the relatively low levels of fibrosis induced by the MCD diet model. Furthermore, the MCD diet does not induce obesity that often accompanies NAFLD in humans. We chose this model because it is reliable and robust—the primary advantage of the MCD diet being that it consistently replicates NAFLD and NASH histological features observed in humans in a relatively shorter feeding time than other dietary models. Nevertheless, future studies that asses the effectiveness of thermal imaging in other models of NASH that induce obesity, elevated fibrosis and lobular inflammation are needed^20^. Furthermore, we did not correlate between thermal imaging parameters and laboratory measurements because we performed the initial measurements, including flow cytometry and blood tests, in a different group of mice than the ones imaged by the thermal camera in the final setting. Finally, thermal imaging has numerous limiting aspects. Notably, it is strongly influenced by environmental factors such as room and working surface temperature, as wells as manual handling of the animal. Accordingly, future experiments should include test settings that standardize these factors as much as possible.

We conclude that non-invasive thermal imaging of the skin covering the liver combined with advanced image processing and machine learning-based analysis is potentially suitable for the monitoring of fatty liver disease in mice. Future development of this technique in humans could lead to important advancements in the field on non-invasive imaging of patients with chronic liver disease.

## Methods

### Methionine-choline deficient diet model

To assess the ability of our thermal imaging technique to detect liver steatosis and inflammation we used a robust model of steatohepatitis in mice fed with a methionine-choline deficient diet^10,11^.

C57BL/6 female mice (8 weeks old) were fed an MCD diet (TD.90262, Envigo) for 6 weeks. Control mice were fed a standard rodent diet (2018SX, Envigo) for the same period of time. Mice were housed in cages (up to 5 mice in each cage) with a 12-h light/dark cycle and were given food and water ad libitum. Whole body weight was measured on a weekly basis. All animal experiments complied with the standards stated in the Guide for the Care and Use of Laboratory Animals (Institute of Laboratory Animal Resources, National Academy of Sciences) and were approved by the Sheba Medical Center Institutional Animal Care and Use Committee.

### Abdominal ultrasound

To assess liver structural changes over time, we performed serial abdominal ultrasound studies with a special small animal ultrasound system (Vevo 2100 Imaging System; VisualSonics, Toronto, Ontario, Canada) equipped with a 22- to 55-MHz linear-array transducer (MS550D MicroScan Transducer, VisualSonics, Toronto, Ontario, Canada). Ultrasound studies were performed on mice fed with MCD/regular diet at week 1, 3 and 5. Light anesthesia was induced by inhalation of 2% isoflurane and 98% O_2,_ and subsequently maintained by 0.5% to 1% isoflurane. All measurements were performed by an experienced technician who was blind to the intervention groups. Qualitative assessment of liver steatosis was determined by an expert clinician in hepatology who was also blind to the experiment.

### Histopathological evaluation

To determine the presence of liver steatosis and immune cell infiltration, we performed histopathological evaluation of the liver at baseline and after 1, 2, 3, 4, 5 and 6 weeks of MCD diet. At each time point, mice (*n* = 4) were euthanized by an over-dose inhalation of isoflurane. Livers were harvested and fixed with 4% buffered formalin (Biolab) and then sectioned into slices. Each slice was embedded in paraffin, sectioned into 5-μm slices, and stained with hematoxylin and eosin (H&E).

### Flow cytometry

To characterize the phenotype of inflammatory cell infiltration to the liver, we analyzed total cell populations isolated from the liver for common mouse monocyte and macrophage markers by flow cytometry. We isolated cells from the livers of mice at baseline and following 1, 2, 3, 4, 5 and 6 weeks of MCD diet (*n* = 4 for each time point). Cells were extracted with an enzymatic digestion mixture as previously described^21^ using 2 cycles of incubation at 37 °C for 10 min. We analyzed the cells by flow cytometry using the fluorescent antimouse antibodies targeted towards CD11 and Ly6C (Biolegend, San Diego, CA, USA). All samples were stained with the related isotype controls. We analyzed cells with a FACS Calibur cytometer (BD Bioscience) using the FlowJo Software (Tree Star).

### Blood work and chemistry panel

To determine the effects of the MCD diet on blood liver enzymes and lipid profile, we collected blood samples for mice at baseline and following 6 weeks of MCD diet. Mice were euthanized by an over-dose inhalation of isoflurane. We sampled roughly 1 ml of whole blood via direct cardiac puncture using a 20G needle. Blood samples were kept on ice for 30 min. To separate the serum, samples were centrifuged at 2000 *g* for 10 min at 4 °C. A chemistry panel was performed to measure the following parameters: total and direct bilirubin, total cholesterol, liver enzymes including alanine transaminase (ALT), aspartate transaminase (AST), alkaline phosphatase (ALP), blood albumin and lactate dehydrogenase (LDH).

### Thermal imaging

We captured thermal images on a weekly basis. Anesthesia was induced as described above for ultrasound studies. To avoid contact-induced interference with skin temperature, we removed hair covering the abdominal region of the mice one day prior to image acquisition. Images were captured using a FLIR One thermal camera device (FLIR Systems, Inc. Wilsonville, OR, USA)^22^. FLIR One utilizes the following functions: a frame rate frequency of 8.7 Hz, an object temperature range of − 20 °C to 120 °C, and thermal sensitivity of 100 mK. The wavelength sensitivity, over which the camera interpolates temperature, is 8–14 µm and the emissivity value considered appropriate for the animal for accurate temperature readings was 0.98. Images were taken at a distance of 10 cm, immediately after the mouse was fixed to an echocardiogram platform. We acquired 3–5 images per mouse which were all processed for downstream analysis.

For intra-abdominal thermal images of the liver in situ, mice after 6 weeks of MCD/regular diet (*n* = 5) were sedated by inhalation of 4% isoflurane and 96% O_2_. The abdomen was opened by gentle dissection in order to expose the liver. A total of 3–5 thermal images of the liver were captured before mice were euthanized by an over-dose inhalation of isoflurane.

### Image processing

The program first read a matrix containing the temperatures in the entire region shown by the thermal image. This temperature map was then displayed and a region of interest (ROI) was selected from it by the user. All textural parameters were computed from the part of the temperature matrix representing this ROI.

First, the algorithm computed the mean temperature in the ROI and its variance.

To compute additional textural parameters using MATLAB commands, the set of temperatures was transformed into non-negative integers with a difference of 0.1 ∘C between adjacent values. The temperatures were first rounded to a precision of 0.1 ∘C (one digit after the decimal point). Finally, the minimal temperature in the ROI was subtracted from all values, and the shifted temperatures were multiplied by 10.

Several textural parameters were extracted from the obtained normalized temperature map. The entropy of the ROI and two moments of the temperature distribution namely its skewness and its kurtosis were computed. Then, the algorithm computed the cooccurrence matrix of the temperature map for the case of a horizontal distance of 20 pixels. From this cooccurrence matrix, the contrast, homogeneity, energy, and correlation were extracted.

To summarize, our algorithm computed and stored nine statistical parameters of the temperature distribution in the selection ROI: four moments of the distribution (mean, variance, skewness, kurtosis), its entropy, and four second-order parameters obtained from the cooccurrence matrix (contrast, homogeneity, energy, correlation).

### Statistical analysis and machine learning techniques

Variables are expressed as median and 95% confidence interval. Specific statistical tests are detailed in the Figure legends. In brief, differences between values were tested by an unpaired t-test. If values were not normally distributed (tested by the D’Agostino Pearson omnibus normality test), we used the non-parametric Mann–Whitney test. We used the Kruskal–Wallis Test along with Dunn’s test for multiple comparisons to assess the significance of measurements between mice at baseline and mice following 1, 2, 3, 4, 5, and 6 weeks of MCD diet.

All statistical analyses were performed with GraphPad Prism version 8.00 (GraphPad Software, La Jolla, CA, USA) and MATLAB software (Mathworks Inc. Natick, MA, USA).

### Machine learning-based analysis

The machine learning analysis was composed of two steps. The first step relied on the computed features from the image processing algorithm, as described above. The dimension of the feature set was reduced by applying a dimensionality reduction method named t-SNE, which compactly describes that data by two new coordinates and allows visualization. The second step was the application of the k-means clustering algorithm in the reduced space. We sought to find *k* = 2 data-driven clusters that separate the mice into two groups. The clustering results were compared with the true mice`s condition.

T-distributed Stochastic Neighbor Embedding (t-SNE) is a nonlinear dimensionality reduction technique, which allows visualization of high-dimensional data by embedding it into a low-dimensional space^23^. The low-dimensional embedding coordinates preserve the data's original probability distribution. In particular, pairs of high-dimensional points that are likely to lie close to one another stay close in the computed embedding. Given the dataset $$X=\left\{{x}_{1},{x}_{2},\dots {x}_{N}\right\},$$ where $${x}_{i}\in {\mathbb{R}}^{D},$$ the similarity between pairs of points is computed by using a Gaussian kernel and denoted by $${p}_{j|i}$$, where $${p}_{j|i}=\frac{{e}^{-{\Vert {x}_{i}-{x}_{j}\Vert }^{2}/2{\sigma }_{i}^{2}}}{\sum_{k\ne i}{e}^{-{\Vert {x}_{i}-{x}_{k}\Vert }^{2}/2{\sigma }_{i}^{2}}}$$ . The kernel's bandwidth $${\sigma }_{i}$$ is adapted to the density around the each point $${x}_{i}.$$ The symmetric distribution is defined by $${p}_{ij}=\frac{{p}_{i|j}+{p}_{j|i}}{2N},$$ where $${p}_{i|i}=0.$$ Let $$Y=\left\{{y}_{1},{y}_{2},\dots ,{y}_{N}\right\},$$$${y}_{i}\in {\mathbb{R}}^{d}$$ denote the low-dimensional map of $$X$$, which reduces the dimension $$D$$ of the original space to $$d\ll D.$$ The goal is to preserve the pairwise distances in the new coordinates. In an earlier algorithm, named Stochastic Nearest Neighbors (SNE)^24^, the conditional probabilities $${q}_{j|i}$$ in the low-dimensional space obtained $${q}_{j|i}=\frac{{e}^{-{\Vert {y}_{i}-{y}_{j}\Vert }^{2}}}{\sum_{k\ne i}{e}^{-{\Vert {y}_{i}-{y}_{k}\Vert }^{2}}}$$ . In t-SNE, a t-students distribution, given by $${q}_{ij}=\frac{{\left(1+{\Vert {y}_{i}-{y}_{j}\Vert }^{2}\right)}^{-1}}{\sum_{k\ne i}{\left(1+{\Vert {y}_{k}-{y}_{i}\Vert }^{2}\right)}^{-1}}$$ is defined instead of the last Gaussian distribution. This modification simplifies the computational complexity. The new coordinates $${\left\{{y}_{i}\right\}}_{i=1}^{N}$$ are computed by minimizing the Kullback–Leibler divergence between the distributions P and Q. This is defined by $$C=KL\left(P||Q\right)=\sum_{i}\sum_{j}{p}_{ij}log\frac{{p}_{ij}}{{q}_{ij}}.$$

The second algorithm that was employed in this work is the k-means clustering technique^25^. Given $N$ data points, the algorithm divided the dataset into $k$ clusters by minimizing the within-cluster variance. K-means is an iterative clustering algorithm. In each iteration k cluster centers (centroids) are computed and each data point is associated with its nearest centroid point forming a new partition to clusters. The process is stopped when the partition to clusters (or, alternatively, the cluster centers) ceases to change.

In this study, the high-dimensional points were the features extracted from the thermal images, as described in “Image processing” denote the dataset of features that was computed from the baseline thermal images by ${X}_{}^{base}=\left\{{x}_{1}^{b},{x}_{2}^{b},\dots ,{x}_{10}^{b}\right\}$ , where ${x}_{i}^{b}\in {\mathbb{R}}^{9}$ , 1 ≤* i* ≤ 10. Each point is a 9-dimesional vector that holds the computed features of a single mouse. In a similar manner, the feature sets that describe the images from weeks 1–6 are denoted by ${X}_{}^{week1},\dots ,{X}_{}^{week6}.$

The t-SNE algorithm was applied to reduce the dimension of the set $${X}^{week2}=\left\{{x}_{1}^{w2},{x}_{2}^{w2},\dots ,{x}_{10}^{w2}\right\}$$ from its original dimension $$D=9$$ to the reduced dimension $$d=2.$$ The resulted coordinates were given by $${Y}^{week2}=\left\{{y}_{1}^{w2},{y}_{2}^{w2},\dots ,{y}_{10}^{w2}\right\}$$ where $${y}_{i}^{w2}\in {\mathbb{R}}^{2}, 1\le i\le 10,$$ and plotted in the top-left image in Fig. 7. Then, the k-means clustering algorithm was applied with *k* = 2 to identify two cluster centroids. The computed centroids were marked by an $$x $$ (see Fig. 7) and the mice associated with each cluster were circled. It can be seen that 8 out of 10 mice were classified correctly. Next, t-SNE was applied on a concatenated dataset, which was formed by unifying the data from $${X}^{week2}$$ and $${X}^{week3}.$$ Each point in this set is of dimension $$D*2=9*2=18.$$ The resulting low-dimensional coordinates $${Y}^{week3}=\left\{{y}_{1}^{w3},{y}_{2}^{w3},\dots ,{y}_{10}^{w3}\right\}$$ are plotted in the top-right part of Fig. 7. The application of k-means to $${Y}^{week3}$$ yielded a correct classification for all 10 mice. In a similar manner, t-SNE was applied to the concatenated sets $${X}^{week2}$$,$${X}^{week3}$$ and $${X}^{week4}$$ (bottom left image in Fig. 7) and last to the concatenated sets $${X}^{week2},\dots , {X}^{week5}$$ (bottom right image in Fig. 7). It can be seen that the k-means clustering algorithm achieved correct classification for all mice in both settings.
